# The Clinical Presentations of Nitrous Oxide Users in an Emergency Department

**DOI:** 10.3390/toxics10030112

**Published:** 2022-02-26

**Authors:** Jhe-Ping Lin, Shi-Ying Gao, Chih-Chuan Lin

**Affiliations:** 1 Lin-Kou Medical Center, Department of Emergency Medicine, Chang Gung Memorial Hospital, Taoyuan 33305, Taiwan; stanley27616159@hotmail.com (J.-P.L.); s78092359@cgmh.org.tw (S.-Y.G.); 2 College of Medicine, Chang Gung University, Taoyuan 33302, Taiwan

**Keywords:** nitrous oxide, emergency department, fast toxidrome, limb numbness

## Abstract

Today, the concomitant abuse of nitrous oxide (N_2_O) and illicit drugs is evident and problematic. However, there are few reports regarding the clinical manifestations of N_2_O users when they present to the emergency department (ED). The purpose of this study was to describe the clinical presentations, the associated illicit substances used in combination, and the outcomes in N_2_O users visiting the ED. This was a retrospective observational cohort study. All N_2_O adult users admitted to the ED at Linkou Chang Gung Memorial Hospital between 2012 and 2020 were included. Demographic variables, clinical symptoms, and examination results were collected from medical records. Univariate comparisons were conducted between pure N_2_O users and combined illicit drug users. A total of 40 patients were included, 24 of which were pure N_2_O users. Limb weakness and numbness accounted for the majority of chief complaints. Neurologic symptoms were the most common clinical manifestations (90%). A more severe ED triage level, faster heart rate, greater agitation, and cardiovascular symptoms were significantly noted in combined illicit drug users. In ED, limb numbness/weakness should arouse physicians’ awareness of patients using N_2_O. Combined use of N_2_O and illicit drugs can cause great harm to health.

## 1. Introduction

Today, the abuse of nitrous oxide (N_2_O) is evident. The lifetime prevalence of N_2_O abuse has been reported to range between 2% and 15.8% among adolescents and young adults [1,2]. The Global Drug Survey (GDS) in 2014 confirmed N_2_O as a very common drug used in the UK and US (38.6% and 29.4% lifetime prevalence) [3]. The abuse of N_2_O remains problematic. For example, there has been a sharp increase in the number of patients with neurological disorders associated with recreational use of nitrous oxide (N_2_O) in China [4]. Unfortunately, there is no epidemiological data of national prevalence of N_2_O abuse in Taiwan. However, due to increasing cases of death after N_2_O abuse found by autopsy, the Taiwan government had added N_2_O to the controlled substance list and forbidden its recreational use by law since 30 October 2020.

Although the use of N_2_O is not associated with significant harm, the adverse effects of N_2_O abuse are well known to include neurologic sequelae such as myeloneuropathy and subacute combined degeneration, as well as psychiatric effects such as psychosis and mood changes [5,6]. Other medical effects such as pneumomediastinum [7], venous thrombosis [8], pulmonary embolism [9], and even death have been reported [10,11]. However, there are few reports regarding the clinical manifestations of N_2_O users when they present to the emergency department (ED). In the ED, physicians do not always know the kinds of substances used by patients due to self-reporting. The concomitant use of N_2_O with other illicit drugs has been reported in the literature [12,13,14,15]. Accordingly, diagnosis may be quite complicated when N_2_O users present to the ED. Therefore, describing the clinical picture of N_2_O users and determining concomitant use of other illicit drugs may help ED physicians to provide better care to patients. Thus, the purpose of this study was to describe the clinical presentations, the associated illicit substances used in combination, and the outcomes in N_2_O users seeking medical help in the ED.

## 2. Materials and Methods

### 2.1. Study Design and Patient Selection

We designed a retrospective observational cohort study. All N_2_O adult users admitted to the ED at Linkou Chang Gung Memorial Hospital (LCGMH) during the study period (2012–2020) were included. LCGMH is an academic teaching hospital located in north Taiwan, with 3700 beds and approximately 14,000 monthly ED visits. The hospital ethics committee (202102441B0, approval date is 27 December 2021) granted approval for this study.

### 2.2. Data Collection

We designed a standardized abstraction form to retrospectively collect all variables from electronic medical records. The following variables were collected: patient’s demographics including age and gender, vital signs at ED triage, ED triage level, patient’s reason for ED visit, positive physical examination of any neurological, psychiatric, cardiovascular, respiratory, or other symptom upon ED admission, patient’s disposition, poison severity score, and outcomes. ED triage level is based on the Taiwan Triage and Acuity Scale (TTAS), a five-level scale used for disease urgency assessment in the study, which is a modification of the Canadian Triage and Acuity Scale (CTAS). There are five levels of urgency: Level 1, resuscitation; Level 2, emergency; Level 3, urgent; Level 4, less urgent; and Level 5, non-urgent [16]. Laboratory variables, including complete blood cell count, as well as serum glucose, sodium, potassium, creatinine, blood urea nitrogen, alanine aminotransferase, and vitamin B12 level (if any) on ED admission, were also recorded. Patients’ urine samples (if any) were sent to be analyzed by the lab to screen (EIA method) for opioids, methamphetamine, MDMA, ketamine, and cannabinoids. The results of the drug screen were also collected.

Examinations such as magnetic resonance imaging, nerve conduction velocity tests, and evoked potentials were arranged after medical ward admission. The results of these examinations were also abstracted.

### 2.3. Statistical Methods

Since the data distribution did not meet the normality assumption, data are expressed as percentages or medians (25th–75th percentiles) unless otherwise indicated. The Mann–Whitney U-test for numerical variables and the chi-squared test for categorical variables were used in univariate comparisons between both study groups: pure N_2_O users and concomitant illicit drug users.

## 3. Results

### 3.1. Demographic Characteristics of ED Patients Intentionally Using N_2_O

A total of 40 patients were included in the study, 24 (12 males) of which were pure N_2_O users with a median age of 25.5 (21.5–29) years. A total of 16 patients (10 males) were concomitant illicit drug users, with a median age of 25 (22–28) years. A total of 11 patients had self-reported N_2_O exposure amount. The average usage frequency of N_2_O was 4.9 ± 2.7 times per week. The average dose of use was 14.9 ± 8.7 bullets per week (one bullet of N_2_O approximately equivalent to 30–40 balloons). The mean duration of N_2_O exposure was 25.1 ± 30.5 months. The demographic characteristics of the patients are listed in Table 1. Many of reasons for the ED visit and various chief complaints were reported by patients. However, limb weakness and limb numbness were the most common, accounting for ~50% of the cohort.

Limb weakness was reported by 29.17%, while limb numbness was reported by 20.83% of pure N_2_O users. On the other hand, the corresponding values were 43.7% and 6.25%, respectively, in concomitant illicit drug users. Neurologic symptoms were the most common clinical manifestations (36 of 40 patients, 90%). Paresthesia and limb numbness (65%), limb weakness (47.5%), and unsteady gait (37.5%) were commonly reported.

### 3.2. Comparison between Pure N_2_O Users and Concomitant Illicit Drug Users

Concomitant illicit drug users presented with a more severe ED triage level (*p* < 0.01) and faster heart rate (92.5 vs. 111 beats/min, *p* = 0.01), as well as more agitation (16.67% vs. 62.5%, *p* < 0.01), and cardiovascular symptoms such as palpitation (4.16% vs. 18.75%, *p* = 0.05) than pure N_2_O users. In contrast, there was no statistically significant difference in other neurologic or respiratory symptoms, as well as in poison severity score (PSS) and patient outcome, between both groups.

On the other hand, half of the patients were either left in the ED for further clinical observation or admitted to the medical ward. A total of 17 patients were left in the ED for observation. Among these cases, seven patients complained of limbs weakness, six patients presented with agitated moods, two patients mentioned abdomen pain, one patient had chest pain, and one patient suffered from headache. These patients had received symptomatic treatment and then were discharged when they became better after ED observation. There were no cases of patient mortality in the study cohort.

Laboratory investigation results are shown in Table 2. Concomitant illicit drug users had a higher white blood cell count and Hb level, while there was no statistically significant difference in serum vitamin B12 level between both groups (270.4, IQR: 152–1772 vs. 491.3, IQR: 200.4–860, *p* = 0.74). Among the 10 patients with elevated white blood cell count, two patients had infections at the same time, and two patients had acute kidney injury and rhabdomyolysis at the same time.

However, eight of 21 (38%) patients initially had a low serum vitamin B12 level (<211 pg/mL). Of the 14 patients checked for serum homocysteine, 12 (85.7%) were found to have a level exceeding the upper limit of 12.42 mmol/L. However, there was no statistically significant difference in serum homocysteine level between both groups. Concomitantly used substances included ketamine (*n* = 11), MDMA (*n* = 5), cathinone (*n* = 4), methamphetamine (*n* = 2), and cannabinoids (*n* = 1).

### 3.3. Findings of Magnetic Resonance Imaging, Nerve Conduction Velocity Tests, and Evoked Potentials

In the study, eight patients were subjected to MRI examination of the cervical spine; three were found normal, four showed symmetrically hyperintense lesions in posterior columns with edema of the posterior spinal cord, and one showed hyperintense lesions in the posterior and posterolateral spinal cord. A total of 17 patients underwent nerve conduction velocity (NCV) tests; 2 were normal, and the other 15 exhibited sensory motor polyneuropathy and demyelinating polyneuropathy. Five patients had their evoked potentials (EP) examined; two were normal, whereas the other three had a peripheral sensory conduction defect with sensory polyneuropathy.

### 3.4. The Severe Poisoned Patients

There were one pure N_2_O user and two concomitant illicit drug users admitted to the ICU. We observed one severe case (a 19-year-old male with a history of pure N_2_O inhalation for 3 years) with severe motor nerve denervation, lower-limb muscle weakness (MRC grade 0–2), and respiratory muscle weakness, as well as subsequent aspiration pneumonia, then acute respiratory failure. The patient was managed with emergent intubation in the ED and admitted to the ICU. Upon recovering from the pneumonia, he was transferred to the rehabilitation ward for neurological sequalae, with a total of 87 days of admission. There were two concomitant illicit drug users admitted to the ICU. The first case was a 21-year-old male who had concomitant abuse of N_2_O, LSD, and cathinone for one day. The patient presented with visual and auditory hallucination then sudden collapse, lying on the ground for an unknown period. When the patient was brought to the ED, he was comatose with hyperthermia, tachyarrhythmia, and hypertension; the initial survey revealed rhabdomyolysis, acute kidney injury, and demanding myocardial ischemia. He ended up with emergency intubation and ICU admission. Another case was a 27-year-old male who abused N_2_O and cathinone in an unknown amount. The patient then had a sudden consciousness change with generalized tonic convulsion. Due to poor consciousness in the ED, he was intubated for airway protection and admitted to the ICU as well.

## 4. Discussion

### 4.1. N_2_O Intoxication

N_2_O can lead to acute intoxication and chronic neurologic or psychiatric sequelae. N_2_O is more water-soluble than oxygen. Once inhaled, it can rapidly diffuse and enter the bloodstream, resulting in dilution of the oxygen volume and decreased oxygen delivery. Upon acute intoxication, it may induce seizures, arrhythmia, respiratory irritation, or even respiratory or cardiac arrest. Inhaling N_2_O in a closed space can lead to asphyxia [17]. Interstitial emphysema and pneumomediastinum secondary to barotrauma after N_2_O inhalation have also been reported [7,18]. Consistent with a previous study, we observed acute consciousness changes, seizures, or respiratory arrest episodes in some patients after substantial N_2_O inhalation in a short period with or without concomitant illicit drug use.

We found that 50% of N_2_O users presented to the ED with limb weakness or numbness, regardless of whether they were pure N_2_O users or concomitant illicit drug users. Therefore, this parameter is important for ED physicians to identify N_2_O use. N_2_O interferes with the vitamin B12 metabolic pathway, leading to reduced recycling of homocysteine to methionine. Methionine is required for nerve myelination. Long-term N_2_O abuse can lead to vitamin B12 deficiency, thus causing demyelination within the central and peripheral nervous systems [19]. Additionally, mental symptoms, anemia, skin changes, and immune disorders are related to chronic N_2_O exposure [20]. Consequently, chronic N_2_O intoxication can lead to severe neurological sequalae such as myeloneuropathy, subacute combined degeneration, peripheral neuropathy, and myelopathy [20]. The most common clinical symptoms included paresthesia, unsteady gait, and weakness, as also reported in [21].

### 4.2. Combined Use of N_2_O and Illicit Drugs

To the best of our knowledge, this was the first study to describe and compare the ED clinical presentations of pure N_2_O users and concomitant illicit drug users. There has been evidence of steadily increasing N_2_O recreational use [4,22]. Almost half of the users in this study indicated the combined use of other drugs. The most common substances used in combination in the study cohort were CNS stimulants, such as ketamine, MDMA, and cathinones. Five of the 16 (31.2%) concomitant illicit drug users were polydrug use (more than three kinds). Toxic coffee packets are a popular form of synthetic cathinones consumed in Taiwan [23]. Due to the polydrug effect, these patients had diverse clinical presentations.

N_2_O inhalation causes an increase in the level of the serum homocysteine. Elevated homocysteine level is an independent risk factor for cardiovascular events [24]. Previous studies have shown that increased homocysteine levels are associated with endothelial dysfunction and increased rates of thrombosis and atherosclerosis [25]. Recreational N_2_O abuse leads to ischemic stroke, myocardial infarction, and venous thrombosis in adolescents which are previously reported [26,27,28,29]. Concerning concomitant illicit drug use, amphetamine and its derivatives are stimulants which have profound effects on the cardiovascular and cerebrovascular systems leading to tachycardia, arrhythmia, vasoconstriction, and hypertension [30]. Amphetamine may cause congestive heart failure, accelerate atherosclerosis, and is associated with myocardial infarction and aortic dissection [31]. Novel psychoactive substances (NPS), such as synthetic cathinones and synthetic cannabinoids, are popular illicit drugs, which also cause severe hazard to health. They pose excessive CNS and cardiovascular effects to abusers, ranging from seizures, paranoid, hallucination, to myocardial infarction, and cardiac arrest [32,33,34]. Accordingly, N_2_O concomitant drug abuse will cause tremendous hazard to the cardiovascular system and cause more toxic effects.

Although the study found no statistically significant differences in poison severity score, disposition, or admission length between both patient groups, a more severe triage level, faster initial heart rate, greater agitation, and more cardiovascular symptoms were significant in concomitant illicit drug users. We believe that, under the effect of multiple stimulants, a “fast toxidrome” was more evident in these patients. Due to apparently abnormal vital signs and a more severe triage level, we assume that ED physicians paid more attention to these patients with immediate and proper treatment. This may explain the lack of a significant difference in outcomes between both patient groups. For example, the higher white blood cell count and Hb level recorded in concomitant illicit drug users may have led ED physicians to administer more intravenous fluid to stabilize the patients.

### 4.3. Examinations of N_2_O Intoxication

Laboratory tests for N_2_O intoxication are not specific. Due to its short half-life and rapid clearance, it is difficult to detect N_2_O in screening tests. A decreased level of vitamin B12 and increased levels of methylmalonic acid and homocysteine can be found in patients with chronic N_2_O intoxication. Due to the poor correlation between serum and tissue levels, functional vitamin B12 deficiency can occur in the presence of a normal serum vitamin B12 level [35]. Some N_2_O users have reportedly self-treated these deficiencies with cyanocobalamin, which can also affect the lab test results [36]. Accordingly, a raised serum level of methylmalonic acid and homocysteine is more sensitive for diagnosis but may not be readily available [5]. In our study, eight of 21 (38%) patients initially had a low serum vitamin B12 level. The prevalence of vitamin B12 deficiency among N_2_O abusers was similar to other studies [37]. A total of 12 of 14 (85.7%) patients presented an elevated homocysteine level. Interestingly, only 4 out of 12 patients who had an elevated homocysteine level showed a low serum vitamin B12 level, highlighting homocysteine as a more sensitive diagnostic tool. In addition to the lab test, diagnosis can be supported by atypical MRI findings or abnormal nerve conduction studies [12]. Characteristically, MRI demonstrates symmetrical T2 hyperintensity within the posterior columns, with variable involvement of the lateral corticospinal tracts [38,39].

### 4.4. Limitations

Our study had some limitations. One of the major limitations was the low patient number. Therefore, interpreting the results, such as prevalence, was not suitable. Therefore, the finding of high prevalence of concomitant abuse in our study may be biased as well, even though our patient number was relatively bigger than or similar to the previous studies [4,6,37,39,40]. Studies of higher evidence should be carried out for a better interpretation of this issue. This study was retrospective and was based on a chart review. Therefore, the study design had inherent limitations such as recall bias, in addition to incomplete chart records or patients concealing their history of N_2_O exposure. The N_2_O inhalation amount and frequency were not recorded in most cases; hence, their relationship with the evaluated parameters was not possible. There is no formal protocol or guideline for N_2_O intoxication management in our hospital. Instead, examination surveys and management are currently based on the ED physicians’ clinical judgement. There is no confirmatory chemical analysis in case of urinary positive screening of drugs of abuse, which may cause false positive results. The pattern of concomitant illicit drug use may differ across countries or regions; therefore, this study’s findings (such as faster heart rate, palpitation, and agitation) of a fast toxidrome should be interpreted cautiously. Due to the limitations mentioned above, we will consider prosecuting a prospective observational study in the future.

## 5. Conclusions

The recreational use of N_2_O is increasing steadily, especially in adolescents and young adults. Almost half of N_2_O abusers are concomitant illicit drug users in the study, and this has become an obvious public health issue. In the ED, limb numbness/weakness should arouse physicians’ awareness of patients using N_2_O. For patients with fast toxidromes, ED physicians should also be aware of the combined use of stimulants.

N_2_O intoxication can lead to severe neurological sequalae, and combined illicit drug use can cause great harm to health. ED physicians should pay additional attention to these patients with immediate and proper treatment.

## Figures and Tables

**Table 1:** Demographic characteristics of ED patients intentionally using N_2_O.

Characteristics Presenting symptoms	Pure N_2_O Users *n* = 24	Concomitant Illicit Drug Users *n* = 16	*p*-Value
Gender (M)	12 (50)	10 (62.5)	0.65
Age	25.5 (21.5–29)	25 (22–28)	0.86
ED triage			<0.01
1	0 (0)	2 (12.5)	
2	4 (16.67)	8 (50)	
3	20 (83.33)	6 (37.5)	
Vital signs			
Respiration rate (/min)	18 (18–19.5)	19 (17–20)	0.74
Heart rate (/min)	92.5 (85.5–105)	111 (97.5–127)	0.01
SBP/DBP (mmHg)	130 (114.5–139)	130 (112–140)	0.91
Body temperature (°C)	36.9 (36.5–37.1)	37.05 (36.7–37.45)	0.37
SpO_2_	97 (96–99)	97 (96–98)	0.39
GCS (median)	15 (15–15)	15 (13–15)	0.11
Reasons for being in ED			0.43
Abdomen pain	2 (8.33)	0 (0)	
Agitation	3 (12.5)	3 (18.75)	
Amnesia	1 (4.17)	0 (0)	
Chest pain	1 (4.17)	0 (0)	
Conscious change	2 (8.33)	4 (25)	
Dyspnea	1 (4.17)	0 (0)	
GTC	1 (4.17)	0 (0)	
Headache	1 (4.17)	0 (0)	
Hemoptysis	0 (0)	1 (6.25)	
Limb numbness	5 (20.83)	1 (6.25)	
Limb weakness	7 (29.17)	7 (43.75)	
Symptoms/signs			
Neurologic symptoms			
Conscious change	7 (29.17)	6 (37.5)	0.84
Unsteady gait	12 (50)	3 (18.75)	0.10
Seizure	1 (4.17)	3 (18.75)	0.98
Headache	1 (4.17)	0 (0)	0.6
Amnesia	4 (16.67)	0 (0)	0.12
Paresthesia/limb numbness	17 (70.83)	9 (56.25)	0.54
Limb weakness	11 (45.83)	8 (50)	1
Psychiatric symptoms			
Agitation	4 (16.67)	10 (62.5)	0.01
Hallucination	6 (25)	4 (25)	1
Dependence	2 (8.33)	5 (31.25)	0.99
Hematology			0.85
Pancytopenia	1 (4.16)	1 (6.25)	
Normal	23 (95.84)	15 (93.75)	
Cardiovascular system			0.05
Shock	0 (0)	2 (12.5)	
Palpitation	1 (4.16)	3 (18.75)	
Normal	23 (95.84)	11 (68.75)	
GI/GU organ systems			0.20
AKI	0 (0)	2 (12.5)	
Abdominal pain	2 (8.33)	1 (6.25)	
Constipation	2 (8.33)	0 (0)	
Urine retention	0 (0)	1 (6.25)	
Normal	20 (83.34)	12 (75)	
Respiratory symptoms			0.42
Dyspnea	1 (4.16)	0 (0)	
Hemoptysis	0 (0)	1 (6.25)	
Respiratory muscle Weakness	1 (4.16)	0 (0)	
Normal	22 (91.68)	15 (93.75)	
Poison severity score (PSS)			0.15
1	9 (37.5)	3 (18.75)	
2	14 (58.33)	11 (68.75)	
3	1 (4.17)	2 (12.5)	
Disposition			0.59
ED observation	11 (45.83)	6 (37.5)	
Admission to ward	12 (50)	8 (50)	
ICU admission	1 (4.17)	2 (12.5)	
Admission length (days)	9 (6–13)	5.5 (3–12)	0.26

**Table 2:** Laboratory investigations of ED patients intentionally using N_2_O.

Lab Variables	Pure N_2_O Users *n* = 24	Concomitant Illicit Drug Users *n* = 16	*p*-Value
White cell count	7 (5.7–8.2)	10.6 (8.4–14.85)	0.01
Hemoglobin	13.1 (11.4–14)	14.3 (12.65–15.2)	0.04
MCV	89.8 (86.3–94.6)	91.55 (86.05–96.4)	0.93
RDW	14.4 (13.8–16.8)	14 (13.45–15.1)	0.25
Platelet	269 (252–373)	282 (201–351.5)	0.53
Blood glucose	109 (100–119)	108 (104–137)	0.76
Na	140 (138–142)	138.5 (136.5–140)	0.21
K	3.7 (3.5–3.8)	3.45 (3.25–4.1)	0.76
BUN	24.4 (23.45–25.5)	22.5 (19.5–24.1)	0.36
Cr	0.77 (0.62–0.94)	0.86 (0.73–1.01)	0.13
ALT	33 (16–51)	31 (19–87)	0.82
Vitamin B12 *	270.4 (152–1772)	491.3 (200.4–860)	0.74
Homocysteine *	33.1 (11.4–57.9)	58 (29.55–98.6)	0.32

* Vitamin B12, *n* = 21; homocysteine, *n* = 14.

## Data Availability

Not applicable.
